# Understanding Genetic Factors in Idiopathic Scoliosis, a Complex Disease of Childhood

**DOI:** 10.2174/138920208783884874

**Published:** 2008-03

**Authors:** Carol A Wise, Xiaochong Gao, Scott Shoemaker, Derek Gordon, John A Herring

**Affiliations:** 1 Seay Center for Musculoskeletal Research, Texas Scottish Rite Hospital for Children, Dallas, TX; 2 Department of Orthopaedic Surgery, University of Texas Southwestern Medical Center, Dallas, TX; 3 McDermott Center, University of Texas Southwestern Medical Center, Dallas, TX; 4 Department of Orthopedics, Kaiser Permanente, San Diego, CA; 5 Department of Genetics, Rutgers University, Piscataway, NJ; 6 Dept. of Orthopaedic Surgery, Texas Scottish Rite Hospital for Children, Dallas, TX, USA

**Keywords:** Scoliosis, genetics, inheritance, genome-wide association.

## Abstract

Idiopathic scoliosis (AIS) is the most common pediatric spinal deformity, affecting ~3% of children worldwide. AIS significantly impacts national health in the U. S. alone, creating disfigurement and disability for over 10% of patients and costing billions of dollars annually for treatment. Despite many investigations, the underlying etiology of IS is poorly understood. Twin studies and observations of familial aggregation reveal significant genetic contributions to IS. Several features of the disease including potentially strong genetic effects, the early onset of disease, and standardized diagnostic criteria make IS ideal for genomic approaches to finding risk factors. Here we comprehensively review the genetic contributions to IS and compare those findings to other well-described complex diseases such as Crohn’s disease, type 1 diabetes, psoriasis, and rheumatoid arthritis. We also summarize candidate gene studies and evaluate them in the context of possible disease aetiology. Finally, we provide study designs that apply emerging genomic technologies to this disease. Existing genetic data provide testable hypotheses regarding IS etiology, and also provide proof of principle for applying high-density genome-wide methods to finding susceptibility genes and disease modifiers.

## INTRODUCTION

“Scoliosis”, derived from the Greek word for crooked, describes a lateral “S-shaped” spinal deformity. Various pathological conditions can present with an associated scoliosis in humans. For example, scoliosis may be secondary to neuromuscular/neurological disease such as Duchenne’s muscular dystrophy, spinal muscular atrophy, or polio. In other cases the spinal deformity is a direct result of vertebral anomalies that are easily visualized by radiography and typically present at birth. However, most scoliosis develops in apparent isolation in an otherwise healthy, pre-adolescent child (Fig. (**1**)). This “idiopathic” form of scoliosis is typically observed during two periods of rapid growth, at infancy and the onset of adolescence. The great majority of cases are of adolescent onset. Although estimates vary, adolescent idiopathic scoliosis (IS) generally affects 2-3% of school age children, and it appears that no ethnic population is spared [1]. Due to the relatively high frequency and potential risks of the disease (see below), many children participate in school screenings for the purpose of early detection [2].

IS is best described as a three dimensional deformity due to rotation of the vertebrae within the curve. Progression of this rotational deformity can ultimately evoke pulmonary compromise [3]. Otherwise the natural history of untreated IS involves increased back pain and spinal osteoarthritis [4,5]. Thus, the central clinical issue in treating IS is anticipating and controlling progression of the deformity. Risk factors for progression in an affected child are well-documented [6]. The most obvious IS risk factor is gender. Affected girls are at least five times more likely to have progressive curves as compared to affected boys, but the reasons for this are unknown [7]. Other risk factors are initial curve pattern, and severity of curvature in relation to remaining growth. Certain curve patterns, e.g. in the thoracic region, are more likely to progress in severity and therefore must be monitored carefully. How growth *per se* provokes disease progression is unclear, but nevertheless the two are highly correlated. Because of this relationship, several measures are typically used to monitor remaining growth in the child with IS. These include menarchal status in girls, bone age, or peak height velocity. In the last case, peak height velocity refers to the point at which the child is at maximum growth, taken from sequential height measurements. Historical data allows estimation of this point, beyond which the child is at lesser risk for curve progression. If curve progression is minimal or controlled through the high-risk period, e.g. by bracing, then surgery may be avoided. However, surgery is warranted for curves that continue to progress (to a threshold of ~50°) before the high-risk period is over [6]. Thus management of IS, from screening and monitoring to intervention, is a considerable health care burden to patients, families, and clinicians.

As will be discussed in this review, many clinical investigations of IS have revealed interesting phenomena associated with the disease, but have failed to produce underlying susceptibility factors or disease modifiers. Thus the aetiological understanding of IS has remained poor (reviewed in [8]). More recently, successes in identifying genetic loci underlying common diseases such as inflammatory bowel disease, psoriasis, age-related macular degeneration, type 2 diabetes, and coronary heart disease [9-18] have generated increasing interest in applying similar genome-wide approaches in large scale studies of IS. Such strategies are appealing in part because they are agnostic to disease aetiology. IS may be particularly amenable to such analyses for several reasons. First, as will be discussed quantitatively in this review, are its significant genetic underpinnings, established by literature going back more than seventy years. Second, IS is routinely phenotyped using standardized objective measures that reduce clinical heterogeneity and thereby may substantially increase power to detect genetic associations. Finally, the onset in childhood enables collection of entire families that can provide information on linkage, association, and inheritance. The recent association of IS susceptibility with variants in the *CHD7 *gene provides proof of concept for these approaches. Here we review IS as a complex genetic disease and compare it to other common diseases in terms of genetic risk. We discuss candidate susceptibility genes and modifier genes in light of recent discoveries. Finally, we present strategies for genome-wide studies to detect haplotypes and copy number variants underlying IS susceptibility. New genetic approaches will reveal insights into disease pathogenesis and may also uncover risk factors for IS susceptibility and progression.

## GENETIC EVIDENCE IN IS

### Is IS genetic?

The recognition of genetic influences in IS is well-documented [19-25]. Familial forms of IS were described as early as 1922 [26]. Since then, reports of multiple twin sets and twin series have consistently shown higher concordance in monozygotic (MZ) compared to dizygotic (DZ) twins (reviewed in [27]). A meta-analysis of these clinical twin studies revealed 73% MZ compared to 36% DZ concordances [28]. Interestingly, in this series there was a significant correlation with curve severity in monozygous twins (P<.0002), but not dizygous twins. No correlation with curve pattern was found, suggesting the importance of genetic factors in controlling susceptibility and disease course, but not necessarily disease pattern. More recently, Andersen *et al*. [29] reported their findings using the Danish Twin Registry. They found 25% proband-wise concordance in monozygotic twins (6 of 44 concordant) compared to 0% concordance (0 of 91) in dizygotic twins, with an overall prevalence of approximately 1%. The lower concordances in both groups as compared with prior results may be explained by differences in study design, specifically, ascertainment in clinics versus by registry, and screening by examination versus questionnaire. Nevertheless the overall trend obtained for all studies suggests strong genetic effects in IS. Interestingly, measured concordances in monozygotic twins were below 100%, reflecting the complexity of disease and suggesting the involvement of as yet unknown environmental or stochastic factors in disease susceptibility.

### How is IS Susceptibility Inherited?

Given that genes contribute to IS, how is disease susceptibility inherited? Autosomal dominant inheritance has been suggested [19-26] from evaluation of single families or small family collections (Fig. (**2**)). X-linked dominant inheritance has been a prevailing theory to explain apparent lack of male-male transmission [22]. However this was disputed after re-evaluation of X ray data from original study subjects [23, 27]). Various studies have found that IS disease risk falls off quickly comparing first-degree relatives of a proband to subsequent generations. Other studies found similar trends [23-25]. Specifically, in their comprehensive population study Riseborough *et al*. reported overall risk to first-degree relatives of 11% compared to 2.4% and 1.4% in second- and third-degree relatives [23]. Interestingly, some but not all studies have found advanced maternal age for mothers of probands with IS [23-25, 30]. These observations may be most consistent with a multifactorial inheritance model involving several to many genes, interplaying with unknown environmental factors. The general consensus gathered from all of this is that, while families with dominant inheritance may exist, IS is generally a “complex” genetic disease that is not easily explained by existing inheritance models.

Here it may be useful to consider insights derived from studies of other so-called complex diseases. For example, *de novo* copy number variation was recently described in ~10% of sporadic cases of autism spectrum disorders, also described as complex genetic disease of childhood [31]. From this observation the authors proposed a model of autism inheritance in which a minority of cases is explained by highly penetrant, dominantly inherited alleles. Remaining cases were explained by sporadic mutations that are highly penetrant in males but not females. In this model, familial (dominantly inherited) cases initiate from sporadic females that are largely unaffected but carry genetically predisposing alleles [32]. Although autism differs from IS in gender distribution and fecundity (IS affected individuals are normal in this regard), as noted IS is described as mostly sporadic and sometimes dominant. Future genomic analysis of IS may shed insight into this issue. In the meantime, as described later in this review, the specification of inheritance model is useful but not requisite for genomic studies aimed at discovering underlying disease alleles.

### What Influences Disease Progression?

As noted, IS disease severity has been correlated in monozygotic twins. This suggests that genetic factors are important not only in IS susceptibility, but also as disease modifiers. However, the variability in disease course typically observed within families suggests that such IS modifier genes are, in general, distinct from susceptibility genes. Environmental factors, perhaps the hormonal milieu, may also influence disease course but this area remains largely unexplored.

### How “Genetic” is IS Compared to other Common and Complex Diseases? 

Familial risk values may be used to estimate and compare the genetic effects across diseases. Prior sibling risk studies of IS have reported 19% and 11.5% of siblings affected for ≥ 10, ≥ 20-degree curves, respectively, compared to population recurrence risks of ≤ 2% [23, 33, 34]. In a cohort of 305 IS families we found 16% of siblings affected (unpublished data) and compared these numbers to the incidence of IS in the general population in order to estimate the sibling risk ratio (λ_s_) for IS. This yielded overall λ_s_ values ranging from 8-23, dependent on curve severity. These values represent significant genetic effects that are comparable to those for other well-described complex genetic diseases such as rheumatoid arthritis (RA), Crohn’s disease (CD), type 1 diabetes (T1D), or psoriasis (Table **1**). The possibility of a major gene contributing to IS, analogous to human leukocyte antigen (HLA) genes in the listed inflammatory diseases, has been suggested but remains unproven [39].

## GENE MAPPING

### Linkage Analyses

Statistical methods may be used to “link” polymorphic biologic markers with disease. Linkage studies may be conducted in a hypothesis-driven fashion, in which markers in specific candidate molecules are tested for linkage with disease. Early linkage studies of IS were driven by the hypothesis that variation in structural components of the spine could be responsible for susceptibility to IS. Polymorphisms in the collagen genes *COL1A1*, *COL1A2*, the fibrillin 1 gene *FBN1*, and the elastin gene *ELN* have been tested in family collections, but the results did not reveal evidence of linkage [40, 41]. The subsequent availability of anonymous polymorphisms across the entire human genome made it possible to conduct systematic searches for IS-linked genes without formulating prior hypotheses about disease aetiology. The first such genome-wide linkage scan was performed in a single family segregating IS in three generations [42]. Subsequent genome-wide scans of IS families and family collections have been reported [43-47]. These have produced possible linkages, three of which are designated IS1, IS2, and IS3 in Mendelian Inheritance in Man (MIM), an official registry of genetic disease loci. IS1 (MIM #181800) was linked to chromosome 19p13.3 in a collection of seven Asian families of southern Chinese descent [44]. IS2 (MIM #607354) was linked to chromosome 17p11 in a single Italian family segregating IS in an apparent autosomal dominant fashion [43]. IS3 (MIM #608765) was linked to chromosome 8q12 in a single extended pedigree and subsequently confirmed in an independent collection of 52 pedigrees [48]. Additionally, genome-wide scans in a collection of 202 families produced varying results dependent on the stratification of the data. Without prior stratification by inheritance model or disease severity, suggestive results were reported for regions of chromosomes 1 and 6. However with stratification other regions became more significant. The authors concluded that regions of chromosomes 6, 9, 16, and 17 are primary regions of interest warranting further analysis [46]. Reported linkages for IS are summarized in Table **2**.

### Cytogenetic Studies

Most genetic diseases are explained by relatively subtle, submicroscopic DNA changes [49]. However, the rare cases with visible cytogenetic alterations can immediately pinpoint causative genes. For example, chromosome 17 rearrangements helped to more precisely localize the NF1 gene responsible for neurofibromatosis [50]. Many chromosomal alterations with phenotypes that include scoliosis have been reported, although most do not appear to re-capitulate idiopathic forms, i.e., without obvious vertebral anomalies or co-existing diagnoses. One family segregating a pericentric inversion of chromosome 8 with idiopathic scoliosis has been reported [51]. Using methods of chromosomal breakpoint mapping, Bashiardes *et al*. [52] found that one end of this inversion disrupted the 8q11.2 gene encoding gamma-1-syntrophin (*SNTG1*), while the other end of the inversion occurred in a gene-free region of 8p23. Subsequent analysis of the *SNTG1* gene in 152 additional IS patients revealed an apparent mutation in DNA samples from three unrelated patients. These changes were not detected in screens of 480 healthy control individuals. These results suggested that rare mutations in *SNTG1* could occur in a small percentage of idiopathic scoliosis patients and left open the possibility that other nearby genes could be important in IS.

### Identifying Susceptibility Genes

Candidate gene studies of IS have been generally unrevealing as noted. In a prior linkage study of 53 families our group identified a large region of chromosome 8q12 linked with IS (locus IS3), apparently near the *SNTG1* gene as described above. Further study revealed that this signal was due at least in part to a different gene encoding the chromodomain helicase DNA binding protein 7 (*CHD7)*, that was both linked and associated with IS [48]. Specifically, multiple single nucleotide polymorphisms (SNPs) in *CHD7* were significantly associated with increased risk of developing disease. That study also produced additional (previously unpublished) observations. First, SNP associations within *CHD7* gene were detected for various inheritance models, underscoring the difficulty in specifying any single model for IS. Also affected members within individual IS families typically differed in severity and pattern of disease, possibly indicating that disease susceptibility and expression were controlled by distinct genetic factors. Alternatively, it is possible that the presence of disease in other affected family members was a phenocopy, or genetically unrelated. When these alternative explanations were tested using the FBAT statistic that considers all affected members in a pedigree, p-values were significant for the SNPs used in the previously published study (Fig. (**3**)). These results supported the conclusion that presence of disease in siblings in this cohort was genetically related, and that separate factors may influence severity/disease course.

## CANDIDATE GENES AND IS AETIOLOGY

IS, at least superficially, appears to involve spine and muscle, and it is therefore perhaps surprising that a neurological pathogenesis is the prevailing opinion to explain disease susceptibility [53]. In this scenario, alterations in vertebral shape and paraspinal muscles may be secondary reactions to an initial neurologic lesion. This model is derived from many observations. For example, scoliosis is often seen in association with neurologic/neuromuscular disease as noted. There is also a well- documented association between apparently isolated scoliosis and spinal cord anomaly such as a syrinx. It is unclear in these cases whether scoliosis occurs secondary to spinal cord deformity, or whether both deformities are secondary to another neurological lesion [53]. Deficits in oculovestibular (visual/hearing) and proprioceptive function have been implicated in some studies of IS patients compared to controls, but other studies have found no differences (reviewed in [8, 34]). In laboratory animals, scoliosis may be induced experimentally or by injury. Small lesions in the pons or periaqueductal gray matter have produced scoliosis in rats and rabbits with low efficiency [8]. Interestingly, removal of the pineal gland from chickens, fish, and bipedal rats produces scoliosis that resembles IS [54-58]. This effect is not seen in quadripeds and suggests that bipedalism/upright posture provokes the deformity. Finally, neurologic underpinnings may be supported by rare “extreme” phenotypes such as the autosomal recessive disease horizontal gaze palsy with progressive scoliosis (HGPPS, MIM #607313). This disease is caused by homozygous loss of function mutations in the ROBO3 gene encoding a transmembrane receptor controlling axon guidance [59]. Brain imaging studies of these patients have confirmed that uncrossed motor and sensory axonal projections are present in the hindbrain. Remarkably, the only clinical manifestations in HGPPS patients are severe scoliosis and absent horizontal eye movement.

How the various neurologic lesions described here could precipitate scoliosis is unclear. In the case of HGPPS patients, the authors hypothesized secondary abrogation of muscle tone and locomotion [59]. An effect on melatonin production has been invoked to explain the effect of pinealectomy in animals, and impaired melatonin signaling has been reported in osteoblasts derived from IS patients [60]. Recently, a case-control analysis found evidence of allelic association for a tagSNP in the melatonin receptor (MTNR1B) promoter region with IS in Asian cohorts [61]. These studies suggest that neuroendocrine pathways, perhaps affecting vertebral growth, may be important in scoliosis. In the case of the IS-associated gene *CHD7*, loss-of-function coding mutations in the gene are known to cause the CHARGE syndrome of multiple developmental anomalies that can include scoliosis. Milder variants leading to a relative reduction of *CHD7* protein has been proposed to explain its association with IS susceptibility [48]. It is interesting to note that in the developing mouse *CHD7* is strongly expressed in primary epithelial tissues, in particular primordial neuronal tissues, with little expression in surrounding mesenchymal tissues. If extrapolated to human postnatal development, this expression pattern suggests IS susceptibility triggered by neurologic pathways rather than *via *direct effects on structural elements, i.e., muscle, bone, and connective tissue.

Despite these observations, lesions of ligamentous spine or paraspinous muscles or cannot be excluded in IS pathogenesis. Whereas histochemical and ultrastructural studies of cartilage, bone, and growth plate in IS patients have been unrevealing [33], an immunohistochemical analysis of the ligamentum flavum revealed some disarrangement of fibers in IS patients compared to controls [62]. Other studies have shown decreased glycosaminoglycan content in the intervertebral discs of IS patients (reviewed in [8]). Histochemical analyses of paraspinous muscles surrounding the scoliotic curve have shown relative hypertrophy and increased electromyographic signaling of type I fibers on the convexity of the curve in IS patients. This was explained as most likely a compensatory response to curve progression [8, 34]. Regarding the bony composition of the spinal column itself, many studies, mostly in Asian cohorts, report generalized osteopenia in IS patients [63-65]. One study found that osteopenia of the femoral neck was a prognostic indicator of curve progression, with an odds ratio of 2.3 [66]. Association between polymorphisms in the estrogen receptor (ESR1) gene and curve severity has been reported, again in Asian IS cohorts, but other studies have not supported this finding [67, 68]. Thus, it is conceivable that genetic variation affecting structural components of the spine may exert quantitative effects on curve progression in IS.

## LARGE SCALE STUDIES TO DISCOVER NEW IS GENES

### Genome-Wide Studies

*A priori*, we do not know what sort of genetic variation will underly IS susceptibility. New methods will eventually enable re-sequencing of candidate regions or entire genomes to identify causative disease alleles [69, 70]. This will be particularly useful for sorting out weak signals produced by genetic heterogeneity. In the meantime, chip-based methodologies are now enabling efficient genome-wide association (GWA) studies to identify disease-associated haplotypes within targeted populations [71, 72]. In some instances, a genotyped SNP itself may be the culprit, while in other instances the genotyped SNPs simply mark common haplotypes carrying disease-associated variants. Either way, genetic associations are measured by comparing large sets of affected cases to healthy controls to detect differences in allele frequencies in the two populations. Unrelated control individuals are relatively easily ascertained but can introduce bias largely due to population substructure [73]. An alternative design is family-based and measures the frequencies that alleles are transmitted from parents to affected offspring. The major benefit of this latter approach is that parents are ideal population controls for their children, but this must be weighed against ascertainment difficulties, particularly for late-onset diseases. With onset early in the second decade of life, family-based designs are ideal for genetic studies of IS. Thus both case-control and family-based designs should prove useful in GWA and follow-up studies of IS.

We previously hypothesized that genes responsible for rare disorders involving scoliosis may be important in “idiopathic” forms of the disease [48]. Some of these rare disorders involve known copy number variation (i.e. duplication/deletion). For example, scoliosis is well-described as part of the phenotype in relatively rare duplication/deletion syndromes such as Charcot-Marie-Tooth disease [74], Smith-Magenis syndrome [75], spinal muscular atrophy [76], Di George syndrome [77] and Prader-Willi syndrome [78]. It is interesting that copy number polymorphisms have been observed within the genomic regions involved in the latter three diseases [79, 80]. Whether common copy number variants are important in scoliosis, particularly IS, is unknown. Methods to detect CNV include array comparative genomic hybridization (CGH) and quantitative analysis of chip-based SNP genotyping [81, 82]. An important consideration here is the possibility that *de novo* CNVs may cause disease as has been detected in autism spectrum disorders. It will be interesting to also consider the possibility that dosage (i.e. copy number) may correlate with some aspect of disease, such as severity or onset, as has been observed, for example, with the CMT-associated 17p duplication [74].

As noted, genetic factors may be important in disease course, i.e. curve progression, in IS. Among the more relevant questions that clinicians have is whether there is some way of predicting whether patients with a certain initial curvature will progress to severe scoliosis requiring intervention, or whether they will not progress significantly, so that no intervention is required. Several statistical methodologies enable use of phenotype and genotype data to investigate such questions. For example, survival analysis methods [83] with longitudinal data enable us to test whether some combination of input variables predicts differing times to "severe" curvature for different patients (e.g., those with a progressive form of disease as compared with those without). In these methods, input variables may include, for example, SNP genotypes, gender, ethnicity, and initial measures of age, curve magnitude, curve pattern, Risser sign, with outcome variable being curve magnitude at a later time point. Also, recently developed mixture modeling techniques [84, 85] ask whether a sample of patients' disease progression trajectories may be decomposed into homogeneous sub-groups of trajectories (e.g., patients with rapid progression in curvature as compared with those with slow progression in curvature), and what input variables classify patients into the different sub-groups. In this way it may be possible to identify combinations of genotypes and phenotypes that classify progressive IS.

For any genome-wide study, however, possible confounding issues must be addressed. The first issue is power and the ability to detect true associations. Power in genetic studies is a function of the disease risk posed by the SNP (genotype relative risk, or GRR), the frequency of SNP variants in a given population, and the frequency of disease in the population [86]. The relatively high frequency of IS (2-3%) in most (if not all populations) and its strong genetic underpinnings relative to other well-studied complex diseases predict good power for genome-wide studies [87]. Estimates of power to detect IS susceptibility alleles are given in Fig. (**4**). The second issue is genetic heterogeneity that can effectively dilute positive signals. Although we do not know *a priori* the source of such heterogeneity, various stratification schemes may be useful to consider in large-scale genetic studies of IS. For example, datasets may be stratified by gender, age at onset, or curve severity in the proband. Regarding gender, male onset is typically later than in females, coinciding with the male adolescent growth spurt, and disease progression continues much later in development, through Risser stage 5 of bone growth [7, 88]. Regarding severity, other linkage studies of IS have found differences when data were stratified by curve severity [45, 46]. Also, increasing estimates of λ_s_ were observed for curves greater than 20° (Table **1**), suggesting greater genetic effect for more progressive disease. For all such strategies, however, it will be important not to unduly burden the study with multiple tests to the point that small gains in statistical significance are lost.

In any planned genetic study of IS it would seem rational to include familial cases of the disease. However, are such cases representative of the more common sporadic cases? As shown in Table **3**, a comparison of sporadic cases to familial cases in one cohort revealed no significant differences in ethnicity, fraction of male probands, age at first presentation or severity in affected individuals (P>0.1 in all instances). Thus families with multiple cases of IS may provide important genetic information that is relevant to the more common sporadic IS patients.

## FUTURE WORK 

In the near term we expect that genomic studies will discover factors with strongest effects on susceptibility to IS, revealing insights into disease pathogenesis. An important question will be how such factors, separately and together, contribute quantitatively to risk of disease (so-called population attributable risk, or PAR). Quantitative analyses and stratification of such datasets may also enable better definition of clinical subtypes within the diagnoses. Identification of genetic factors underlying these diseases will enable further research enhancing the prospect for alternative, less invasive therapies.

## Figures and Tables

**Fig. (1):**
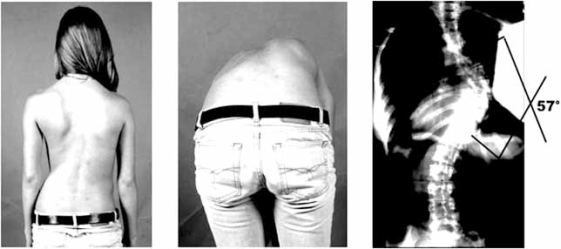
**IS in an adolescent female.** Physical examination reveals a rib hump on forward bending (left-most panels). X rays on the right reveal a right thoracic curve measuring 57 degrees but without vertebral or other structural anomalies.

**Fig. (2):**
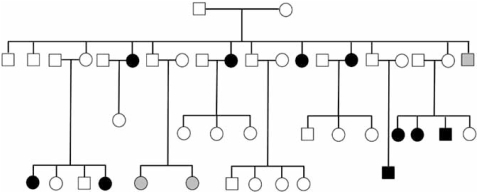
**IS inheritance in an extended pedigree.** Blackened, clear, and gray symbols represent affected, unaffected, and mildly affect (<15° curvature) individuals. This family exhibits possible autosomal dominant inheritance of IS with reduced penetrance.

**Fig. (3):**
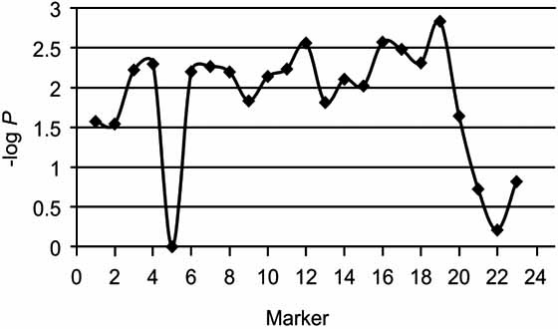
**FBAT analysis of 53 families with idiopathic scoliosis.** Negative log of the associated P-value is plotted on the Y axis versus ordered polymorphisms in the CHD7 gene along the X axis.

**Fig. (4):**
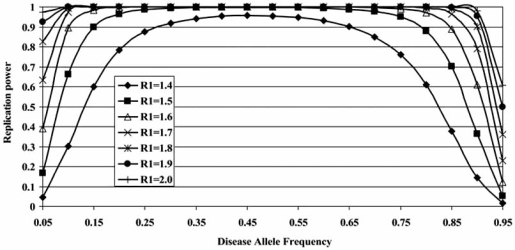
**Power to detect and replicate association as a function of genotype relative risk (*R*_1_) and disease allele frequency.** The analysis assumes a two-stage design as described by Skol *et al*. [89], although modified so that power is calculated for TDT rather than case-control sampling. The total sample size is 2,100, with 700 trios included in stage 1 and 1,400 trios included in stage 2. Power is computed assuming that the disease locus operates in a multiplicative fashion and for a genome-wide significance of .10.

**Table 1. Calculated Sibling Risk Ratios for IS are Comparable to Other Well-Studied Complex Genetic Diseases:** We compared the frequency of IS in siblings of probands with IS to the frequency of IS in the general population. This produced an estimated sibling risk ratio (λs) of 8 for curves ≥ 15° and 23 for curves ≥ 20°

Disease	Prevalence	λs	Reference
RA	.01	2-17	[35]
CD	.001	10	[36]
T1D	.007	15	[37]
Psoriasis	.02	4-11.5	[38]
IS (≥10°)	.03	8	Unpublished observations
IS (≥20°)	.005	23	Unpublished observations

**Table 2. Reported Linkages for IS:** Regions proposed to harbor IS susceptibility genes are given by cytogenetic location, position relative to known polymorphisms, and MIM designation. References a-g refer to citations [42-48], respectively.

Chromosomal Region	OMIM Locus	Flanking Loci	Ref.
6p		D6S1051-D6S1017	a, b
6q		D6S1053-D6S1021	b
8q	IS3	D8S1477-D8S279	c
9q		D9S938-D9S934	b
10q		D10S1222-D10S212	a
16q		D16S764-D16S2624	b
17p	IS2	D17S974-D17S1294	d
18q		D18S1357-D18S1371	a
19p	IS1	D19S1034	e, f
Xq		GATA144D04-GATA172D05	g

**Table 3. Clinical and Demographic Comparison of Multiplex and Simplex IS Families with Atleast 4 Members:** Ethnicity was determined by self-report of parental origin. Cobb angles were maximum measurements recorded prior to correction (caw, jah previously unpublished data).

	Multiplex	Simplex
% non-European	9	8
% male probands	18	17
Age at 1^st^ presentation (std dev)	11.7 (+/-2.9)	12.1 (+/-2.8)
Cobb angle (median)	40.3° (39°)	42.1° (42°)
